# First things first: A late robotic approach to anamnesis, in a patient with a thymus hematoma

**DOI:** 10.1259/bjrcr.20200017

**Published:** 2020-06-15

**Authors:** Maria Grazia R. Manco, Alessia Francavilla, Gian Maria Ferretti, Giuseppe Guglielmi, Marco Taurchini

**Affiliations:** 1 Department of Radiology, University of Foggia, Foggia, Italy; 2 Unit of ThoracicSurgery, IRCCS Fondazione Casa Sollievo Della Sofferenza, San Giovanni Rotondo, Foggia, Italy

## Abstract

Anterior mediastinal masses are generally asymptomatic until they grow and compress surrounding structures. Chest X-rays only suggest a mediastinal abnormality and contrast-enhanced CT scan and MRI are necessary for a better definition of the lesion. The classification of the anterior mediastinal masses is based on their etiology and it is sometimes a challenge to have an accurate differential diagnosis based only on radiological examinations: therefore, only the histopathological examination makes the correct diagnosis. Surgeons generally agree that symptomatic masses or those with progressive growth should undergo surgical resection. We report a case of an accidental finding of an organized thymic hematoma in a 46-year-old female. At first totally asymptomatic, the hematoma was misdiagnosed for a thymic cyst and resected when it increased in size and compressed surrounding mediastinal structures. A detailed anamnesis highlighted a minor thoracic trauma which turned out to be the cause. Retrosternal hematoma generally grows several months after trauma and initial stabilization; therefore, it is mandatory to include an organized hematoma in the differential diagnosis of the retrosternal neoformations.

## Introduction

The anterior mediastinum is defined as the thoracic part lying behind the sternum and before great vessels and pericardium. It extends from the diaphragm, inferiorly, to the thoracic inlet, superiorly, and contains thymus, mediastinal fat, lymph nodes, internal mammary arteries and veins.^1^

Mediastinal cysts are well-marginated round lesions that contain fluid and are lined with epithelium. They represent 12–30% of all primary mediastinal masses, more than half of which are located in the anterior compartment.

Mediastinal masses are most frequently asymptomatic, but they may cause cough, dyspnea, chest pain, Horner syndrome and other symptoms due to the compression they apply on surrounding structures.

In the diagnostic process, it is of primary importance to take into account chest radiography findings suggestive of a mediastinal abnormality, since chest X-rays are the most common imaging examination performed. Although small lesions may not be visible, large lesions often may manifest with loss of the normal mediastinal contours or a mass in the retrosternal space in lateral chest projection. Therefore, chest X-rays can only be considered a screening examination.

Once an abnormality is identified at chest radiography, CT with intravenous contrast agent is the first choice imaging modality. MRI and PET/CT scan can be used in selected cases and offer additional information that can further refine the differential diagnosis.^2,3^

Since it is difficult to make an accurate diagnosis relying on radiological examinations only, surgeons generally agree that symptomatic cysts or those with progressive growth should undergo surgical resection.

We present the case of a patient with a neoformation of the anterior mediastinum, treated with a feasible, safe and mini-invasive thoracic surgical technique for the mediastinum pathology: robotic-assisted thoracoscopic surgery (RATS).

## Case presentation

We report the case of a 46-year-old female, with a family history of Familiar Dilative Cardiomyopathy, admitted to the Thoracic Surgery Department of our Hospital for an unusual massive neoformation of the anterior mediastinum. The patient had no significant comorbidities and was a smoker of five packs of cigarettes/year.

## Investigations

In 2018, the patient was subjected to a contrast-enhanced CT of head and neck because of persistent hemicrania. Accidentally, the upper thorax section images documented the presence of a neoformation of the anterior mediastinum. A thoracic high-resolution CT (HRCT) was then performed and documented the presence of a fluid oval lesion (77×47 mm) in the right-middle mediastinum, positioned between the right anterolateral tracheal wall and the carina and with no contrast enhancement of its thin walls. Therefore, the diagnosis of a cystic neoformation of the thymus was suggested and in consideration of the Hounsfield Unit (HU) scale of the cyst content, it was defined as a complicated cyst. Differential diagnoses also led to the suspect of a thymic cystic neoplastic lesion.

Laboratory tests, performed to rule out cyst superinfection, were in the normal ranges with negative phlogosis indices. The possibility of characterizing the mediastinal neoformation through fine needle aspiration biopsy (FNAB) was excluded due to its cystic component. The removal of the neoformation through a sternotomy was therefore suggested, but the patient refused. The female was therefore offered a close follow-up.

**Figure 1.**
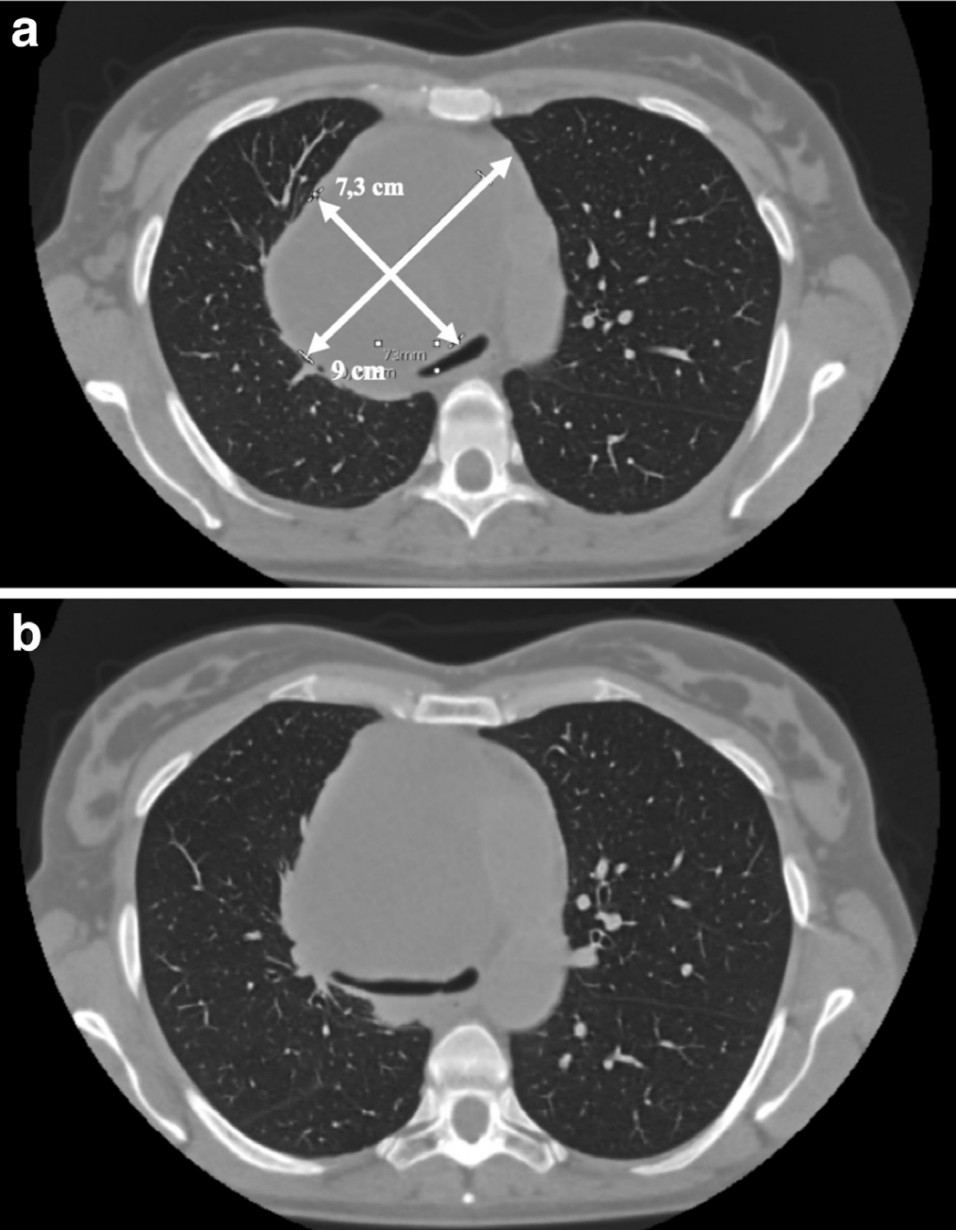
Thorax CT scan without contrast of the first days of January 2019. (a) axial view of the neoformation of the dimensions of 90x73 mm with compression of the trachea (b) axial view of the neoformation with compression of the right main bronchus

In January 2019, both chest X-ray and chest CT documented an increase in size (91×73 mm) (Figure 1). After a few days, the patient presented at the emergency room reporting the onset of dyspnea under stress, chest pain and cough. She was urgently submitted to an echocardiogram that documented compression of the right and left atria by an external mass. Laboratory tests were in the range of normality.

**Figure 2.**
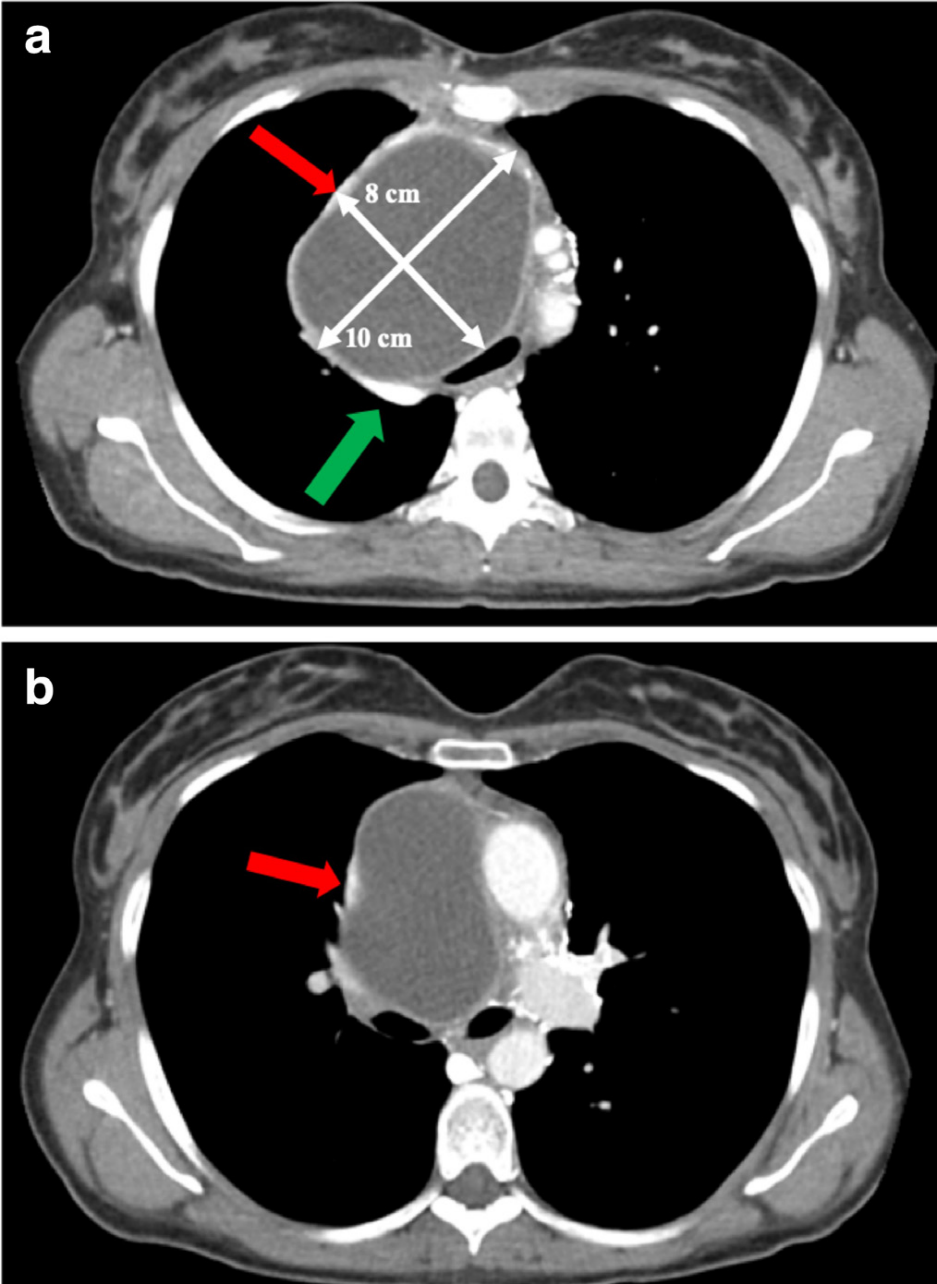
Preoperative thorax CT scan with contrast. Red arrow indicating the compression of the SCV, a green arrow indicating the azygos vein. (a) axial view of the neoformation (10×8 cm) with c.e. of its walls compressing the trachea, (b) axial view of the neoformation with compression of the SCV and of the right main bronchus

**Figure 3.**
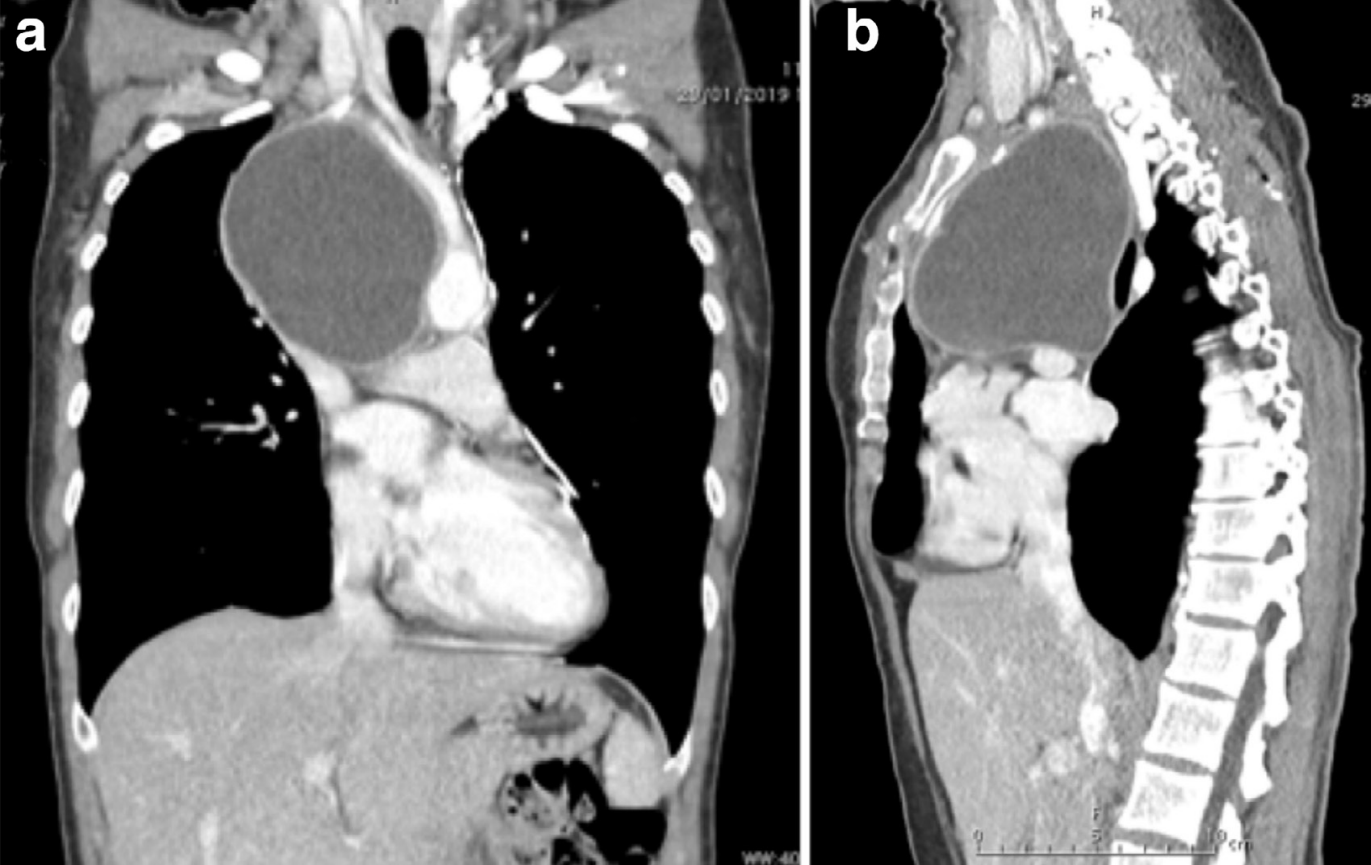
Preoperative thorax CT scan with contrast. (a) coronal view shows the lesion compressing the right pulmonary artery and its closeness to the aortic arch and the brachio-cephalic trunk, (b) Sagittal view of the lesion slightly compressing the right pulmonary artery.

Another thorax CT examination was then performed, revealing extension of the lesion in the upper and middle mediastinum (10×8×11 cm) with contrast enhancement of its walls. Medially, the lesion was close to the aortic arch and the brachio-cephalic trunk (Natsis type-I); cranially, it was close to the right subclavian vein; caudally, it was adjacent to the right pulmonary artery, which appeared slightly compressed. The mass was also very close to the superior cava vein (SCV) and the anonymous veins, sharply compressed and dislocated (Figures 2 and 3). The main compressive effect was the involvement of the right main bronchus and the right pulmonary artery with minimal atelectasis of the upper pulmonary lobe on the same side.

In consideration of the increase in the size of the mediastinal neoformation and the initial symptomatic compression of the mediastinal structures, the patient was hospitalized and prepared for surgery.

## Differential diagnosis

The main diagnostic hypotheses were complicated thymic cyst or germ cell tumor with a cystic component, but different types of anterior mediastinum masses exist and may have similar clinical features. Therefore, it is important to distinguish one from another:

- **Bronchogenic cysts**: result from abnormal ventral budding of the tracheobronchial tree during embryological development. Their CT attenuation values can vary from water-to-soft tissues. Most of them are localized in the subcarinal or right paratracheal region;
- **Neuroendocrine tumors**: composed of small, uniform and round cells. The most common one is thymic carcinoid, the onset of which is observed in Cushing Syndrome;
- **Mediastinal thyroid**: enlarged gland distorted by multiple nodules and surrounded by a fibrous capsule. It causes displacement and compression of the trachea;
- **Germ cell tumors**: on CT, teratoma contains a prominent cystic component, small dense localized areas of calcification, fat or mixed low-density material;
- **Thymolipoma**: composed of lobules of fat and thymic tissue;
- **Thymoma**: composed of thymic epithelial cells mixed with lymphocytes. Almost half of the patients with thymoma suffer from Myasthenia Gravis;
- **Thymic cysts**: they fall into three main categories, congenital, acquired and neoplastic. On CT, congenital ones appear as well-defined water-attenuation masses with imperceptible walls;
- **Pericardial cyst**: pericardial cysts are rare entities and comprise 7% of all mediastinal masses. They are commonly congenital, but traumas, bacterial or parasitic infections, pericarditis and cardiac surgery may also result in the formation of pericardial cysts.^4,5^
- **Lymphomas**: 15% of the mediastinal masses are lymphomas (approximately 60% are Hodgkin lymphoma, 20% are non-Hodgkin lymphoma and 20% other subtypes). Chest CT generally documents a lobulated or smooth soft tissue attenuating mass of the anterior mediastinum with paratracheal involvement. Presentation with low-density cystic areas are common.^6^

## Treatment

As a first step, to decide the best following surgical approach, exploration of the anterior mediastinum was performed using the right robotic approach with the Da Vinci Si. On 30th January 2019, the patient underwent robotic surgery under general anesthesia.

The exploration of the anterior mediastinum confirmed the presence of giant neoformation of brownish color starting from the thymus, with dislocation of the surrounding structures. In consideration of the dimensions and to avoid the execution of a sternotomy, a biopsy of the mass was obtained and sent for a fresh frozen examination. The tissue resulted in negative for neoplasm and documented the presence of fibrous tissue containing blood cells. Therefore, an additional utility incision was performed at IV intercostal space to allow the decapitation of the mass.

Its fluid content was aspired (about 400cc of old blood like chocolate color material) and the neoformation was then cautiously moved from the vena cava and the pericardium, but it presented tenacious adhesion to the thymic tissue and the pericardium in its most caudal portion. For this reason, the lesion floor remained in place and was not removed due to its non-neoplastic nature. After the removal of the neoformation, all the structures that were found to be dislocated returned to their natural anatomical sites.

**Figure 4.**
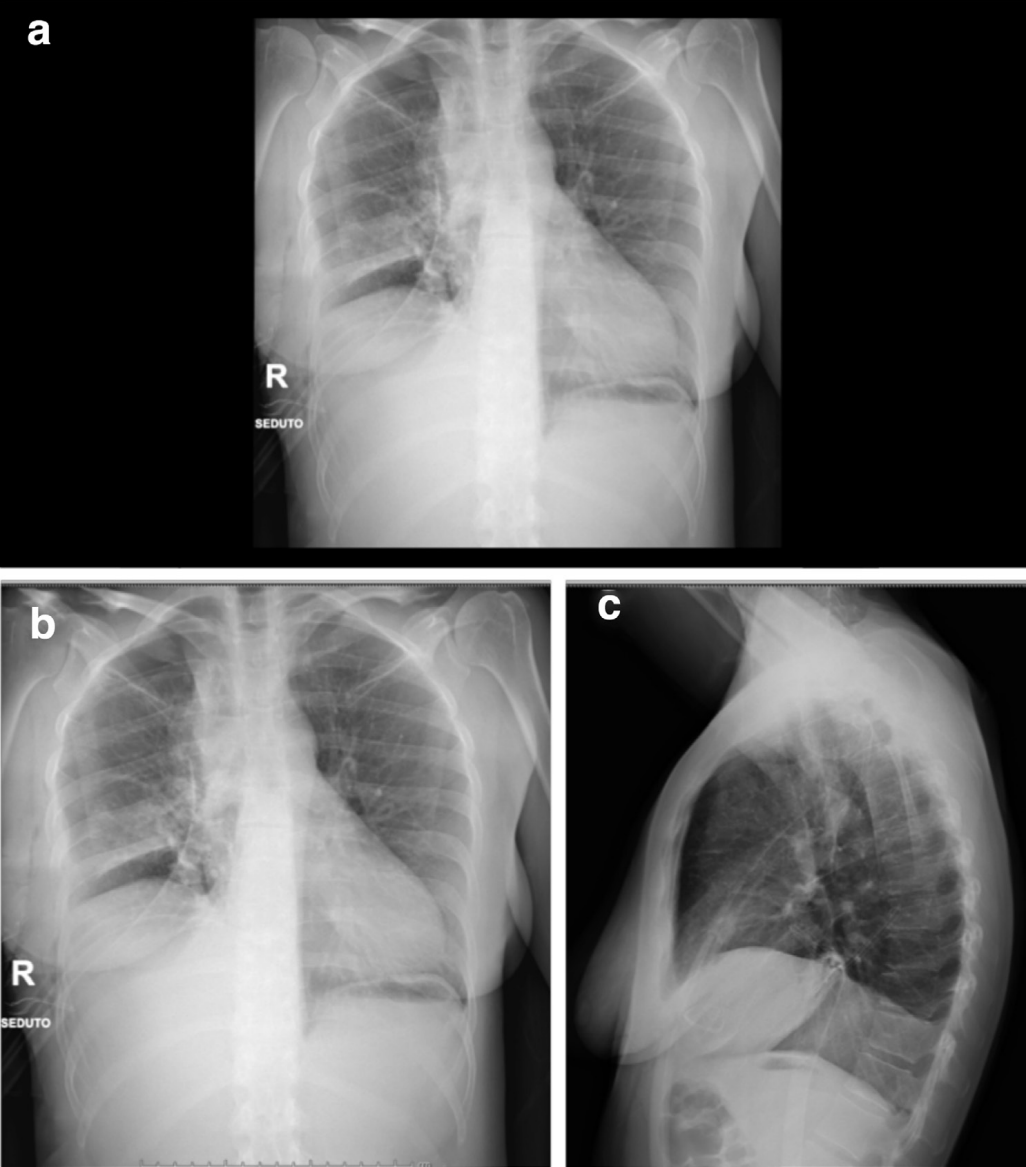
Postoperative chest X-Rays that document the mediastinum in the correct anatomical location. (a) First postoperative day, (b) and (c) Fourth postoperative day preremoval of pleural drainage.

The postoperative course was regular with a resolution of the symptoms. Following radiological examinations documented on-axis mediastinum (Figure 4) and pleural drainages were removed on the fourth postoperative day. The patient was discharged on the fifth post-operative day.

## Outcome and follow-up

The definitive histological examination documented fibro-adipose tissue with hemorrhagic content covered by hemosiderophages, compatible with outcomes of chronic hemorrhaging, mixed with lymphoid tissue containing thymic lymphocytes (CD 3+, TdT+). The presence of cystic epithelium was not documented, therefore conclusive diagnosis of chronic intrathymic hemorrhage was made.

The patient was therefore called for the communication of the outcome of the histological examination and she was asked to refer to any previous thoracic traumas. She reported having a small thoracic trauma years ago caused by a car crash, that did not require hospitalization or performing in-depth examinations but that was followed by a few days of retrosternal pain.

Our final diagnosis was an organized thymus hematoma.

**Figure 5.**
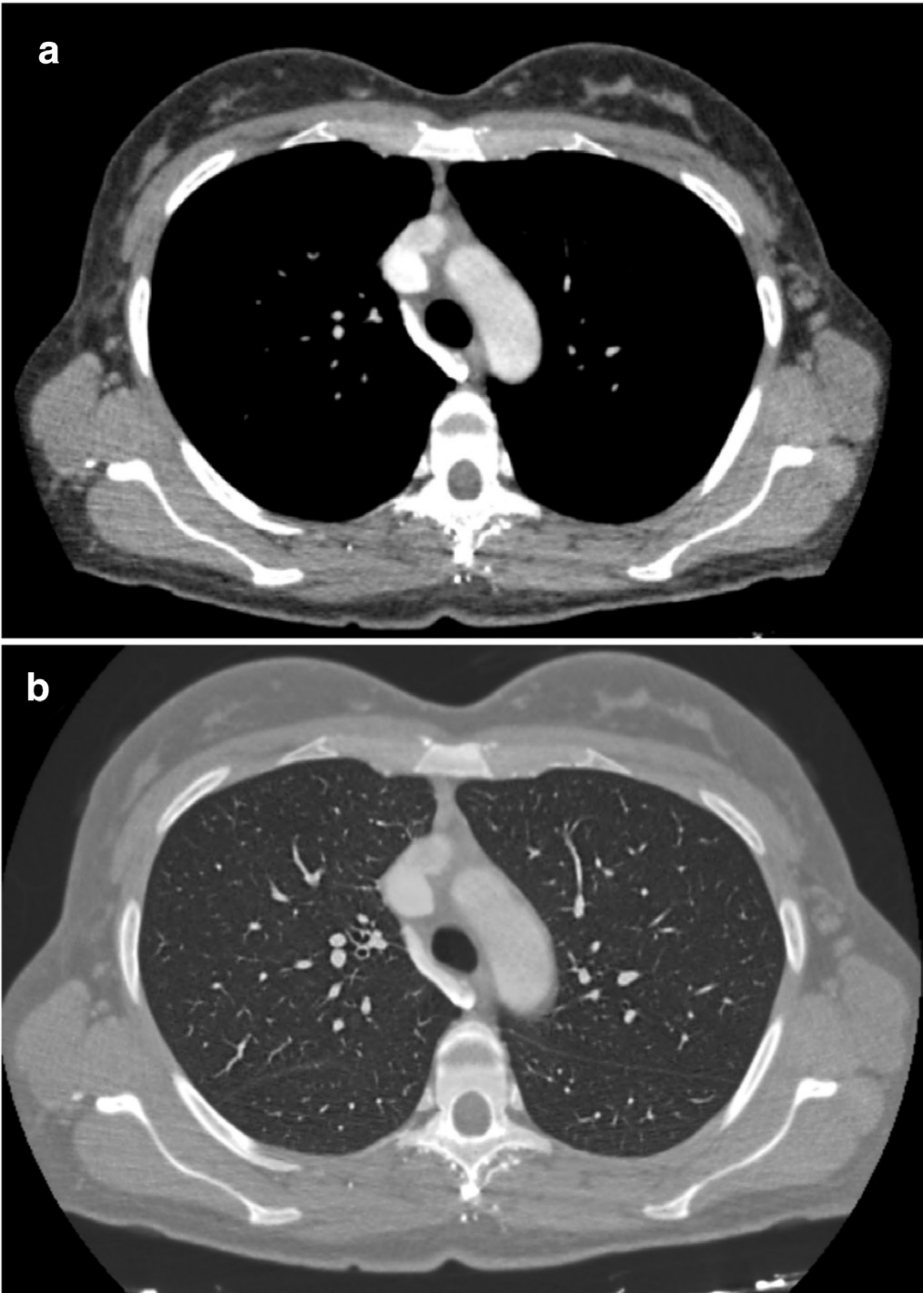
Thorax CT scan with contrast at 6 months after surgery. (a) and (bB) axial view of the mediastinum without sign of recurrence.

Follow-up at 3 months with chest X-ray and at 6 months with thorax CT with contrast documenting no recurrence of the disease (Figure 5).

## Discussion

Despite the advancement of technological innovation, the most important action that a doctor has to accomplish is an accurate anamnesis. In our case, if a detailed medical history had been collected, an organized hematoma would have been probably taken into consideration for the differential diagnosis. On the other hand, it must be considered that the clinical trend of the retrosternal hematoma was particular because of its slow increase in size, which occurred several months after the trauma. An examination that, if carried out, would probably have given clinicians additional imported data is MRI. MRI could have provided important information regarding the content of retrosternal collection and probably allow a distinction between complicated thymic cysts and hematoma. Today, minimally invasive surgery represents a valid option. The robotic-assisted thoracic surgery (RATS), as demonstrated in many articles, allows an ideal approach to the diseases of the anterior mediastinum.^7–10^

In our clinical case, robotic surgery allowed an optimal exploration of the mediastinum without needing to perform a sternotomy. The robotic technique approach first allowed to make a diagnosis and subsequently to assess the relationship between neoformation and mediastinal structures. Thanks to a high-definition three-dimensional view and more precise and flexible robotic arms, the surgeon can assess the actual operability of the patient and decide to continue the surgery with a minimally invasive robotic technique.

## Learning points

- The medical history and the physical examination of the patients are fundamental instruments in the hands of a medical doctor.
- The first investigation is a chest X-ray, that helps detecting a loss of normal mediastinal contours, followed by chest contrast-enhanced CT, which represents the best choice to study mediastinal masses.
- Hematoma should be included in the differential diagnosis of retrosternal neoformations, considering its characteristic growth, even several months after the initial stabilization following a trauma.
- In case of indeterminate pathologies of the anterior mediastinum, an MRI could be very useful, providing complementary and additional information to the CT scan.
- In cases of undetermined or doubtful pathologies of the anterior mediastinum, a minimally invasive exploratory robotic approach must be evaluated in order to obtain a diagnosis and to assess the effective operability of a patient before proceeding with a sternotomy.
